# How do poverty types reshape the effect of political identity on prosocial behavior? The chain mediation role of emotion regulation and compensatory effect of cultural heritage education

**DOI:** 10.3389/fpsyg.2026.1849695

**Published:** 2026-05-15

**Authors:** Wei Zhang, Yuxin Wang, Guoping Wang, Zhongbao Tang

**Affiliations:** 1 School of Marxism, Wannan Medical University, Wuhu, China; 2 School of Anesthesiology, Wannan Medical University, Wuhu, China; 3 School of Humanities and Management, Wannan Medical University, Wuhu, China; 4 School of Marxism, Jiangnan University, Wuxi, China

**Keywords:** conservation of resources theory, cultural heritage education, emotion regulation, medical students, political identity, poverty types, prosocial behavior

## Abstract

**Introduction:**

Based on four types of poverty defined by the combination of objective socioeconomic status and subjective economic affluence, this study examined the chain mediation mechanism through which political identity is associated with medical students’ prosocial behavior via emotion regulation strategies and further explored whether the effects of cultural heritage education (as measured by red music knowledge) differed across poverty types.

**Methods:**

A survey was conducted among 1,070 medical students, measuring political identity, emotion regulation, prosocial behavior, red music knowledge, objective socioeconomic status, and subjective economic affluence. To control for the confounding effect of grade level and ensure that all participants had received the same cultural heritage education curriculum, the primary analyses were restricted to second-year students (*n* = 782). Cluster analysis was employed to identify poverty types, and chain mediation models and multi-group analysis were used to examine the pathways across the different types.

**Results:**

(1) Political identity positively predicted prosocial behavior, with a significant indirect effect mediated by the chain mediation pathway of cognitive reappraisal and expressive suppression. (2) Cluster analysis identified four types of poverty: subjectively advantaged, dual-disadvantaged, objectively advantaged, and dual-affluence. (3) Path analysis revealed significant differences across poverty types: In the objectively advantaged type, two independent pathways (political identity direct effect and cognitive reappraisal → expressive suppression) additively predicted prosocial behavior, reflecting a resource additive effect; in the dual-disadvantaged type, the chain mediation pathway was not significant, but red music knowledge indirectly predicted prosocial behavior through political identity; in the subjectively advantaged type, subjective affluence positively predicted prosocial behavior through cognitive reappraisal; in the dual-affluence type, only cognitive reappraisal directly predicted prosocial behavior.

**Discussion:**

Political identity is associated with prosocial behavior through a chain mediation of cognitive reappraisal and expressive suppression. This mechanism exhibits qualitatively distinct patterns across the four poverty types, manifesting as four corresponding resource effects: substitution, compensation, additive, and dilution. These findings provide mechanistic explanations for the prosocial function of political identity, extend the application boundaries of the conservation of resources theory, and offer empirical evidence for targeted educational interventions tailored to students experiencing different poverty types.

## 1 Introduction

Cultivating responsible citizens remains a central challenge for education systems worldwide. Systematic educational practices aimed at fostering students’ civic knowledge, skills, and attitudes—or civic education—are regarded as the primary means of achieving this goal (United Nations Educational, Scientific and Cultural Organization, 2015). Among the many objectives of civic education, the cultivation of political identity is particularly crucial. Political identity is defined as an individual’s sense of belonging and commitment to their political community—including the nation, ethnicity, institutions, and values. It is believed to foster a sense of social responsibility that transcends personal interests, thereby promoting altruistic behavior (Simić et al., 2022). For medical students, this prosocial orientation is particularly important: it is not only a core manifestation of humanistic care in medical practice but also the psychological foundation for building trust between future doctors and patients (Burks and Kobus, 2012).

### 1.1 Political identity and prosocial behavior: a social identity perspective

Political identity is a multidimensional construct. It is grounded in ethnic-racial or cultural identity (Umaña-Taylor et al., 2014) and extends further to include identification with political institutions and value systems (Erikson, 1968), forming three intertwined core dimensions: emotional attachment, cognitive beliefs, and behavioral tendencies (Huddy, 2001). Social identity theory posits that individuals derive part of their self-concept from group membership and tend to exhibit positive attitudes and prosocial behavior (PB) -that is, behaviors intended to promote others’ safety and well-being, such as cooperation, care, comfort, assistance, and defense (Dunfield, 2014; Tomasello, 2018) toward in-group members (Tajfel and Turner, 1979). This theory lays the foundation for understanding the relationship between political identity and PB: when individuals incorporate a political community (including the nation, ethnic community, institutions, and value systems) into their self-concept, they develop a sense of responsibility toward the community and its members, as well as prosocial tendencies. Political identity encompasses not only emotional attachment but also a set of values and behavioral norms internalized through socialization.

The prosocial functions of political identity involve multi-layered psychological mechanisms. First, at the motivational level, the need for belonging inherent in identity drives individuals to safeguard the well-being of the group, leading to a psychological fusion of group and personal interests (Deci and Ryan, 2000). Second, at the normative level, identity is accompanied by the acceptance and internalization of core norms such as mutual aid and cooperation within the ingroup (Grusec and Davidov, 2010). Finally, at the emotional level, positive emotions such as pride and a sense of belonging evoked by political identity create favorable psychological conditions for the emergence of PB (Eisenberg and Miller, 1987). From the perspective of moral foundation theory, political identity may also activate individualizing moral foundations (e.g., care, fairness), thereby further promoting PB toward in-group members, particularly when the political community emphasizes shared moral values (Ahluwalia-McMeddes et al., 2025).

Empirical research supports the positive association between political identity and PB. In Western contexts, social identity can motivate PB among individuals with different political orientations, with processes such as cultural adaptation and racial socialization playing central roles (Streit et al., 2020; Knight et al., 2016; Schwartz et al., 2007). In Eastern contexts, relevant research has also validated this relationship, suggesting that emotional factors may play an important role (He et al., 2025). Moreover, recent meta-analyses have confirmed the reliability and generalizability of PB measures across diverse populations and settings (Reig-Aleixandre et al., 2023; Badenes-Ribera et al., 2023), supporting the robustness of the measurement approach used in the present study.

However, it should be noted that the prosocial function of political identity is not unconditional. Research has shown that when political identity takes the form of nationalism (rather than patriotism), defensive national identity, or collective narcissism, it may instead exacerbate intergroup bias, xenophobia, or even intergroup conflict (Gao F. et al., 2024; Gronfeldt et al., 2023; Yogeeswaran and Dasgupta, 2014). Moreover, the meaning and effects of political identity vary across cultural contexts and ideological backgrounds—as evidenced by research on cross-cultural differences between collectivist and individualist societies (Triandis, 1995; Markus and Kitayama, 1991)—and do not follow a uniform psychological pattern in all societies (Uysal et al., 2025; Park and Warner, 2024).

Nevertheless, how political identity translates into PB remains underexplored. Uncovering this psychological mechanism is a prerequisite for understanding the boundary conditions of this relationship. Specifically, three key questions arise: (1) Through what kind of psychological process does political identity affect PB? (2) Are these processes universal or group-specific? (3) How do external factors (e.g., economic background, culture, and education) regulate this transformation?

### 1.2 Mediating role of emotion regulation: an emotion regulation perspective

According to emotion regulation theory, emotion regulation refers to the process by which individuals influence their own emotional experiences, intensity, and expression, with cognitive reappraisal (altering the cognitive interpretation of a situation) and expressive suppression (inhibiting emotional expression) serving as two core strategies (Gross, 2015). As a leading theoretical framework in the field, Gross’s Extended Process Model (Gross and John, 2003; Richards and Gross, 2000) distinguishes these two strategies: cognitive reappraisal is an adaptive strategy that effectively modulates emotional experiences, fostering positive emotions and cognitive resources; conversely, expressive suppression is a maladaptive strategy that, while controlling emotional expression, may deplete cognitive resources and impair social interaction. This model has been widely applied in recent studies to explain emotion regulation processes and their impact on mental health across diverse populations (O'Brien et al., 2025; Cai and Samson, 2025). Recent meta-analytic evidence has systematically confirmed a significant positive association between emotional intelligence (EI) and PB (Cao and Chen, 2025). Notably, emotion regulation ability is a core component of emotional intelligence (Peña-Sarrionandia et al., 2015). Given that political identity inherently involves emotional experiences (e.g., belonging, pride) and that emotion regulation is a core component of EI, it is theoretically sound to examine emotion regulation strategies as the mediating mechanism through which political identity translates into prosocial behavior.

The broaden-and-build theory of positive emotions suggests that positive emotions broaden cognitive and behavioral resources and build enduring personal resources (Fredrickson, 1998; Fredrickson, 2001); thus, the positive emotions inherent in political identity (e.g., belonging, pride) may activate cognitive reappraisal, which then strengthens positive emotions and promotes PB. This inference is supported by empirical evidence: children and adolescents with higher emotion regulation abilities demonstrate better empathy, moral decision-making, and PB (Fabes et al., 1994; Hein et al., 2018; Song et al., 2018; Wilke and Goagoses, 2023).

Collectively, these findings identify emotion regulation as a core psychological mechanism linking internal states to external prosocial expressions. Importantly, this mechanism may be particularly consequential for individuals facing economic adversity. Poverty not only constrains material resources but also imposes a psychological burden that impairs cognitive function, increases stress, and depletes the cognitive resources necessary for effective emotion regulation (Haushofer and Fehr, 2014; Park et al., 2024). Under such conditions, individuals may rely more heavily on expressive suppression as a default strategy, which paradoxically exacerbates emotional distress and hinders prosocial engagement. Conversely, the ability to use cognitive reappraisal—reframing stressful situations—becomes even more critical for maintaining psychological health and fostering PB in lower socioeconomic status contexts (Troy et al., 2017).

### 1.3 Poverty profiles as boundary conditions: a conservation of resources perspective

The conservation of resources (COR) theory posits that individuals possess a fundamental tendency to strive to acquire, retain, protect, and nurture the resources they value; psychological stress arises from the potential or actual loss of resources, as well as from failing to obtain expected gains after investing resources (Hobfoll, 1989). The “resources” are defined as things that individuals value or means that help them obtain what they value, encompassing material goods, conditions, individual characteristics, and energy (Hobfoll, 2001). Furthermore, their value is subjective and situational (Halbesleben et al., 2014).

Based on COR theory, this study makes the following core proposition: the influence of a resource is directly proportional to its scarcity. When a resource is scarce, its value is amplified, generating compensatory demand; when a resource is abundant, its effect is diluted; and functional equivalence and substitution relationships may exist among different resources (Hobfoll, 2002). Poverty may moderate the political identity–PB link through both structural and psychological pathways. Structurally, lower SES restricts material resources and social opportunities, directly reducing the capacity to engage in costly prosocial actions (Wu et al., 2025). Psychologically, scarcity captures attention, depletes cognitive bandwidth, and amplifies the perceived value of limited resources (Civai et al., 2024). This core proposition is further supported by several principles of COR theory: the loss-preference principle, the resource investment principle, the initial resource effect, and the resource fleet and channel principles (Hobfoll, 1989; Baumeister et al., 2001; Hobfoll et al., 2018).

A recent meta-analysis suggests that the relationship between social class and PB exhibits high heterogeneity (Wu et al., 2025), which may stem precisely from different combinations of objective and subjective factors. Research in the Chinese context similarly presents a complex picture (Gao H. et al., 2024; Hu et al., 2025; Liu et al., 2023; Wang et al., 2021; Zheng et al., 2025), further highlighting the limitations of focusing on a single dimension and suggesting that objective resources and subjective perception are often separated, with their different combinations producing unique effects (Mullainathan and Shafir, 2013; Robinson and Piff, 2017).

The present study integrates the above perspectives and proposes the concept of “poverty types”—combining objective SES with subjective economic affluence (SEA) to identify four distinct resource contexts. Based on COR theory and related research (Smith et al., 2012), we anticipate the following four resource effects: resource dilution effect (the diminished impact of a single resource when resources are abundant), resource compensation effect (external resources serving a compensatory function when resources are scarce), resource substitution effect (functional equivalence among different resources), and resource additive effect (the additive contribution of multiple independent pathways when resources are inconsistent). These anticipated effects will be empirically examined in this study.

### 1.4 Exploratory role of cultural heritage education

Within this framework, cultural heritage education, as a key vehicle for fostering political identity, may play a crucial role in the resource compensation effect (Carlo et al., 2025), particularly for the dual-disadvantaged type. Relevant studies indicate that such education can promote the development of cultural identity (Douglass and Umaña-Taylor, 2016), but its effectiveness is influenced by factors such as the implementation context (Walton and Yeager, 2020). Moreover, from the perspective of COR theory, the impact of cultural heritage education may depend on an individual’s resource context—for types experiencing dual resource scarcity, it may serve a compensatory function by reinforcing political identity; whereas for types with relatively abundant resources, its marginal effects are relatively limited. In this study, cultural heritage education is treated as an exploratory independent variable, examining whether it indirectly influences PB through political identity. Following previous research (He et al., 2025), we operationalized it as red music knowledge, a measurable component of cultural heritage education, which assesses the ability to identify the composition periods of 12 well-known revolutionary songs. As an exploratory measure, it primarily captures students’ cognitive mastery of specific cultural symbols and has inherent limitations in fully representing the broader construct of cultural heritage education. Therefore, findings related to this variable should be interpreted with caution. Based on the above reasoning, we propose a corresponding hypothesis, which is presented in Section 1.5.

### 1.5 Research questions and hypotheses

It should be noted that preliminary analyses revealed significant grade-level differences in the core variables. To control for the confounding effect of grade level and ensure that all participants had received the same cultural heritage education curriculum, subsequent core analyses were restricted to sophomore students. Therefore, the hypotheses given below are tested on the sophomore subsample (*n* = 782).

In summary, this study proposes the following research questions and hypotheses:

*Research Question 1*: Does political identity influence PB through the serial mediation of cognitive reappraisal and expressive suppression?

*Hypothesis 1: Political identity positively predicts PB, and this relationship is serially mediated by cognitive reappraisal and expressive suppression.*

*Research Question 2*: Does the above serial mediation pathway vary across poverty profiles (combinations of objective SES and subjective economic affluence)?

*Hypothesis 2a: (Resource Substitution Effect): Among the subjectively advantaged type (low SES, high SEA), subjective economic affluence will substitute for political identity, promoting PB via cognitive reappraisal.*

*Hypothesis 2b: (Resource Compensation Effect): Among the doubly disadvantaged type (low SES, low SEA), the serial mediation pathway will not be significant; however, external cultural resources will play a compensatory role by reinforcing political identity.*

*Hypothesis 2c: (Resource Additive Effect): Among the objectively advantaged type (high SES, low SEA), the serial mediation pathway will not be significant. Instead, political identity will directly predict PB, and cognitive reappraisal will indirectly predict PB via expressive suppression, indicating an additive effect.*

*Hypothesis 2d: (Resource Dilution Effect): Among the doubly affluent type (high SES, high SEA), the serial mediation pathway will not be significant; PB will primarily stem from emotion regulation abilities.*

*Research Question 3*: Does cultural heritage education (indexed by red music knowledge) play a differentiated role across poverty profiles?

*Hypothesis 3: Cultural heritage education indirectly promotes PB through political identity only in the doubly disadvantaged type, with no significant effects in other types.*

Given the measurement limitations of red music knowledge as an indicator of cultural heritage education, findings related to Hypothesis 3 should be interpreted as exploratory.

## 2 Methods

### 2.1 Participants and design

An anonymous cluster sampling survey was conducted from June to July 2025 among first- and second-year medical students at a medical university in Anhui, China. The survey was administered as a group activity in classrooms by the researchers and faculty members of the course. The questionnaire consisted of two parts: the core variables questionnaire was completed via an online survey platform, while the political identity questionnaire was administered independently in paper form offline. To ensure data consistency between the online and offline components, respondents were required to create a unique password (e.g., the last four digits of their birthdate plus the last four digits of their mobile phone number) and enter it on both questionnaires; researchers used this to link the data.

A total of 1,300 questionnaires were distributed. After excluding those that were incomplete, invalid, or could not be matched, 1,070 valid questionnaires were obtained, resulting in a valid response rate of 82.31%. The age range of the valid sample was 17–22 years (*M* = 19.60, *SD* = 0.97). Among these, 782 valid questionnaires were from sophomore students, constituting the core sample for subsequent analysis. It is important to note that higher-year medical students were not included in this study, because they typically enter clinical practice and no longer receive the unified cultural heritage education curriculum, which is delivered only in the first 2 years. The specific composition of the sample is shown in Tables 1, 2.

**Table 1:** Test of differences between first- and second-year students on core variables (*M* ± SD, *N* = 1,070).

Variable	Freshmen (*n* = 288)	Sophomores (*n* = 782)	Mean difference	*t*	95%CI
Political identity	82.9 ± 4.47	82.98 ± 4.98	−0.01	−0.02	[−0.66,0.65]
Cognitive reappraisal	30.14 ± 4.93	29.30 ± 5.10	0.84	2.41*	[0.16,1.52]
Expressive suppression	17.00 ± 4.05	16.06 ± 4.05	0.94	3.36***	[0.39,1.49]
Prosocial behavior	101.04 ± 11.49	99.27 ± 11.39	1.77	2.25*	[0.22,3.31]
SEA	0.10 ± 0.94	−0.04 ± 1.02	0.14	2.04*	[0.01,0.28]
SES	0.16 ± 0.95	−0.06 ± 1.01	0.21	3.11**	[0.08,0.35]

SEA and SES z-scores are standardized within the full sample (*N* = 1,070). Other variables are the raw scores. *p < 0.05, **p < 0.01, ***p < 0.001.

**Table 2:** Descriptive statistics and correlation matrix for core variables (*N* = 782).

Variable	*M* ± SD	1	2	3	4	5	6	7	8	9	10	11	12
Control variables													
1. Gender^a^	–	1	–	–	–	–	–	–	–	–	–	–	–
2. Age	19.82 ± 0.91	−0.122**	1	–	–	–	–	–	–	–	–	–	–
3. Household registration^b^	–	0.038	0.031	1	–	–	–	–	–	–	–	–	–
4. Are you a student leader?^c^	–	0.140**	0.04	−0.061	1	–	–	–	–	–	–	–	–
5. Have you received any scholarships or financial aid?^d^	–	0.207**	0.008	0.057	0.232**	1	–	–	–	–	–	–	–
Core variables													
6. Red music knowledge score	6.60 ± 3.45	−0.015	−0.045	−0.010	0.020	0.059	1	–	–	–	–	–	–
7. SEA^e^	−0.04 ± 1.02	0.006	−0.019	−0.193**	0.013	−0.150**	−0.032	1		–	–	–	–
8. SES^e^	−0.04 ± 1.00	0.038	−0.091*	−0.439**	0.063	−0.214**	−0.005	0.330**	1	–	–	–	–
9. Political Identity	82.98 ± 4.98	0.101**	0.005	−0.006	0.051	0.072*	0.081*	0.008	0.017	1	–	–	–
10. Cognitive reappraisal	29.30 ± 5.10	0.023	−0.01	−0.072*	0.065	0.005	0.027	0.256**	0.103**	0.072*	1	–	–
11. Expressive suppression	16.09 ± 4.05	−0.196**	0.008	0.018	−0.067	−0.119**	−0.046	−0.075*	0.034	−0.015	0.224**	1	–
12. Prosocial behavior	99.27 ± 11.39	0.034	−0.036	−0.074*	0.145**	0.065	0.070	0.131**	0.052	0.164**	0.409**	0.153**	1

a Gender: 0 = male, 1 = female.

b Residence: 0 = urban, 1 = rural.

c Student leadership role: 0 = no, 1 = yes.

d Scholarships/grants: 0 = no, 1 = yes.

e SES and SEA are standardized scores (z-scores) based on the full sample (*N* = 1,070).

Categorical variables are reported as percentages (see main text); continuous variables are reported as means and standard deviations. Pearson correlation coefficients are shown below the diagonal. *p < 0.05, **p < 0.01.

Comparisons between the sophomore subsample (*n* = 782) and freshmen (*n* = 288) showed no significant difference in student leadership status (*χ^2^* = 5.45, *p* = 0.142); however, significant differences were found in age (*t* = 15.84, *p* < 0.001), gender (*χ^2^* = 5.77, *p* = 0.016), household registration status (*χ^2^* = 7.59, *p* = 0.006), and receipt of scholarships or financial aid (*χ^2^* = 86.56, *p* < 0.001). To control for these potential attrition biases, the subsequent analyses included age, gender, household registration status, and receipt of scholarships or financial aid as covariates.

The survey covered 16 out of 19 medical-related majors, and the three omitted majors were either newly established, had very small enrollments, or substantially overlapped with the surveyed majors. The sophomore subsample represented approximately 26% of the total sophomore population (*N* ≈ 3,000). *A priori* power analysis using G*Power 3.1 (Faul et al., 2009) (linear multiple regression, medium effect size *f*^2^ = 0.15, *α* = 0.05, power = 0.80, number of predictors = 9) indicated a required sample size of 118. Thus, the final sample (*n* = 782) was adequately powered.

This study was conducted in accordance with the ethical principles of the Declaration of Helsinki. The Medical Ethics Committee of Wannan Medical University approved this study and the format of the participant consent form.

### 2.2 Measures

Detailed reliability and validity indicators for all scales are presented in the Supplementary Table S1.

#### 2.2.1 Political identity scale

The Political Identity Scale developed by Wang and Zhao (2024) measures individuals’ sense of belonging and commitment to their political community. The questionnaire comprises 17 items with two dimensions: the nation and ethnicity, as well as political systems and values. The scale uses a 5-point Likert scale (1 = strongly disagree, 5 = strongly agree), with higher scores indicating a higher level of political identity. In this study, the internal consistency reliability coefficient for the total scale was 0.94.

#### 2.2.2 Emotion regulation questionnaire

The Emotion Regulation Questionnaire (Gross, 2002) consists of 10 items and includes two dimensions: cognitive reappraisal (6 items) and expressive suppression (4 items). A 7-point Likert scale was used (1 = strongly disagree, 7 = strongly agree); higher scores on each dimension indicate more frequent use of the corresponding emotion regulation strategy. In this study, the internal consistency reliability coefficients for cognitive reappraisal and expressive suppression were 0.90 and 0.81, respectively.

#### 2.2.3 PB questionnaire

The Prosocial Tendencies Measure (Kou et al., 2007) consists of 26 items and includes six dimensions: openness, anonymity, altruism, compliance, emotionality, and urgency. A 5-point Likert scale was used (1 = not at all like me, 5 = very much like me); higher scores indicate a stronger tendency toward PB. In this study, the internal consistency reliability coefficient for the total scale was 0.95.

#### 2.2.4 Cultural heritage education questionnaire

As a symbolic vehicle of China’s revolutionary culture, red music holds a central position in cultural heritage education. To assess students’ cognitive mastery of this important form of cultural heritage, we selected 12 representative and well-known red music pieces. Multiple experts were invited to evaluate the representativeness of the music, ensuring that the selected music spanned different historical periods and were widely sung, thereby guaranteeing content validity. To measure respondents’ knowledge of the composition periods of the 12 well-known music pieces, one point was awarded for each correct answer, with a scoring range of 0–12 points; a higher score indicates a greater mastery of red music knowledge. The internal consistency reliability coefficient for the 12 items was 0.82 in this study.

#### 2.2.5 Objective SES scale

An SES scale was adopted as a tool to measure objective poverty. The SES of university students was assessed using the methodology outlined in the 2009 Programme for International Student Assessment (PISA) (OECD, 2010) and Xu et al.’s (2024) study. Participants were required to report information regarding their parents’ educational attainment, occupations, and the number of household possessions. These data were then scored as follows: parental educational attainment was assigned scores based on the specific duration of each educational level in China, using the PISA standard as a reference. (1) Specifically: incomplete elementary school was assigned 3 points; elementary school, 6 points; junior high school, 9 points; vocational high school/senior high school, 12 points; junior college, 15 points; and university or graduate school and above, 16 points. (2) Scores ranged from 1 to 10 points. Parental occupation used occupational codes from the China Family Panel Studies, converted to the International Standard Classification of Occupations (ISCO-88), and then matched to the corresponding occupational prestige index (Li, 2005). Participants were also required to report on 14 types of household resources, such as whether their household owns a computer or has a private room; each item was assigned 1 point, with a total score ranging from 0 to 14. The scores for these three variables were calculated to yield a composite SES score. The specific formula is SES = (*β*_education_ × *Z*_education_ + *β*_occupation_ × *Z*_occupation_ + *β*_possessions_ × *Z*_possessions_) / *E*, where *β* represents the factor loading for each variable, *Z* represents the standardized score for each variable, and *E* is the eigenvalue of the first factor. A lower SES score indicates a lower SES of the college student’s family and a higher degree of objective financial scarcity.

#### 2.2.6 SEA questionnaire

Adapted from Griskevicius et al.’s (2011) Perceived Resource Availability Scale, this 10-item scale measures individuals’ subjective perceptions of their financial situation. A 7-point Likert scale was used (1 = strongly disagree, 7 = strongly agree), with items 2, 4, 5, 6, 7, and 8 reverse-scored; higher scores indicate a greater subjective sense of financial well-being. In this study, the internal consistency reliability coefficient for this scale was 0.89. To maintain consistency with SES and facilitate subsequent poverty type clustering, the SEA scores were standardized in the full sample (*N* = 1,070).

### 2.3 Statistical analysis strategy

We utilized SPSS 26.0 and its PROCESS macro (version 4.1) for data analysis. The specific analytical strategy was as follows:

1) Descriptive statistics and correlation analysis: The mean, standard deviation, and Pearson correlation coefficients for each variable were calculated to preliminarily examine the degree of association among variables, providing a foundation for subsequent analysis.
2) Identification of poverty types: Based on the standardized SES and SEA within the full sample, K-means cluster analysis was then performed on the full sample to identify poverty types. The number of clusters (four) was specified based on theoretical expectations, and the Calinski–Harabasz criterion supported this choice. The resulting cluster centers were then used to assign poverty type membership to the sophomore subsample (*n* = 782) for subsequent path analyses. K-means was chosen over LPA for the following reasons: (1) with only two continuous indicators, K-means directly corresponds to the theoretical four-quadrant classification and is computationally simple; (2) the Calinski–Harabasz criterion supported the four-cluster solution; (3) LPA as a robustness check() also yielded a four-class solution that closely matched the K-means typology (three of four classes matched); and (4) the full-sample clustering showed high agreement with the subsample clustering (Kappa = 0.724). Thus, our poverty typology is not an artifact of the clustering algorithm (see Supplementary material for detailed LPA results).
3) Testing the chained mediation model: We employed Model 6 (Hayes, 2013) from the PROCESS macro developed by Hayes to test the chained mediation effect whereby political identity influences PB through cognitive reappraisal and expressive suppression. The theoretical order of mediators follows Gross’s extended process model: cognitive reappraisal is an antecedent strategy that modulates emotions before they arise, whereas expressive suppression is a response-focused strategy. Hence, we specified cognitive reappraisal as the first mediator and expressive suppression as the second. We used the bias-corrected percentile bootstrap method with 5,000 repetitions to calculate the 95% confidence interval for the indirect effect. If the confidence interval does not include 0, the mediation effect is significant. For significant indirect effects, we computed partially standardized effect sizes and assessed multicollinearity using VIF.
4) Step-by-step path analysis: A step-by-step strategy was employed to examine differences in the relationships among core variables across the four subgroups: first, correlations among variables were tested; second, regression analysis was conducted on significantly correlated variables; and finally, based on the regression results, corresponding mediation path models were constructed and tested using PROCESS macro Models 4 and 6 within each subgroup to assess how pathways differed across poverty types.

All statistical analyses employed two-tailed tests with a significance level of *α* = 0.05; *p* < 0.05 was considered statistically significant.

## 3 Results

### 3.1 Common method bias test

Common method bias was tested using Harman’s one-factor test (Podsakoff et al., 2003). All measurement items were included in an unrotated exploratory factor analysis. The results showed that the first factor explained 21.59% of the total variance, which is below the critical threshold of 40%, indicating that there is no serious common method bias.

### 3.2 Preliminary analysis: test for grade-level differences

In the valid sample (*N* = 1,070), an independent samples *t*-test was used to examine differences between first- and second-year students on each core variable; the results are shown in Table 1.

First-year college students (freshmen) scored significantly higher on cognitive reappraisal (*t* = 2.41, *p* < 0.05), expressive suppression (*t* = 3.36, *p* < 0.001), PB (*t* = 2.25, *p* < 0.05), SEA (*t* = 2.04, *p* < 0.05), and objective SES (*t* = 3.11, *p* < 0.01). This result may reflect the “positive bias” (Stanley-Clarke et al., 2024) commonly observed among freshmen during their initial period of enrollment, as they are full of anticipation for college life and more likely to exhibit positive psychological and behavioral tendencies in their self-reports. By contrast, the second-year college students (sophomores) have experienced campus life for longer, leading to more stable scores across all indicators, which better represent the typical level of the college student population. No significant differences in political identity were found across grade levels (*p* > 0.05), indicating that this core variable exhibits stability across grades. Therefore, as explained in Section 1.5, the subsequent core analyses were conducted only on the sophomore subsample (*n* = 782) to control for grade effects and ensure curriculum consistency, thereby eliminating the “novelty effect’” among freshmen.

### 3.3 Descriptive statistics and correlation analysis of the core sample

The core analysis sample (sophomores, *N* = 782) had a mean age of 19.85 years (SD = 0.913, range 18–22), with 256 male (32.7%) and 526 female (67.3%) participants; 255 students (32.6%) held urban household registration, while 527 (67.4%) held rural registration; 244 students (31.2%) held leadership positions in student organizations at various levels, while 538 (68.8%) did not; 391 students (50%) received scholarships or financial aid. The survey covered 16 out of 19 medical-related majors. The means, standard deviations, and Pearson correlation coefficients for the remaining variables are presented in Table 2.

The correlation matrix revealed the following results.

Relationships between control variables and core variables: Gender was significantly positively correlated with political identity (*r* = 0.101, *p* < 0.01), indicating that female students have a stronger sense of political identity than male students; gender was significantly negatively correlated with expressive suppression (*r* = −0.196, *p* < 0.01), indicating that female students use expressive suppression strategies less frequently.

Age was significantly negatively correlated with SES (*r* = −0.091, *p* < 0.05), indicating that younger students had higher SES scores. Household registration was significantly negatively correlated with SEA, SES, cognitive reappraisal, and PB (*r* = −0.193, −0.439, −0.072, −0.074, *ps* < 0.05), indicating that students with urban household registration scored higher on these four variables.

Student leadership status was significantly positively correlated with PB (*r* = 0.145, *p* < 0.01), indicating that students serving in leadership roles exhibited higher levels of PB.

Receipt of scholarships and financial aid was significantly negatively correlated with SEA, SES, and cognitive reappraisal (*r* = −0.150, −0.214, −0.119, *ps* < 0.01) and significantly positively correlated with political identity (*r* = 0.072, *p* < 0.05), indicating that students receiving scholarships and financial aid have lower objective and subjective economic resources, use less expressive suppression, and exhibit higher political identity.

Relationships among core variables: Red music knowledge scores were significantly and positively correlated with political identity (*r* = 0.081, *p* < 0.05). Sense of affluence was significantly positively correlated with SES, political identity, and PB (*r* = 0.330, 0.256, 0.131, *ps* < 0.01), and significantly negatively correlated with expressive suppression (*r* = −0.075, *p* < 0.05). SES was significantly positively correlated with cognitive reappraisal (*r* = 0.103, *p* < 0.01). Political identity was significantly positively correlated with cognitive reappraisal and PB (*r* = 0.072, 0.164, *ps* < 0.05). Cognitive reappraisal was significantly positively correlated with expressive suppression (*r* = 0.224, *p* < 0.01). Both were significantly positively correlated with PB (*r* = 0.409, 0.153, *ps* < 0.01).

In addition to the original covariates (gender, age, household registration status, and receipt of scholarships or financial aid), student leadership status was included as a control variable. All correlations were below 0.44, indicating no multicollinearity concerns.

### 3.4 Chain mediation effect of political identity on PB: full-sample test

**Figure 1:**
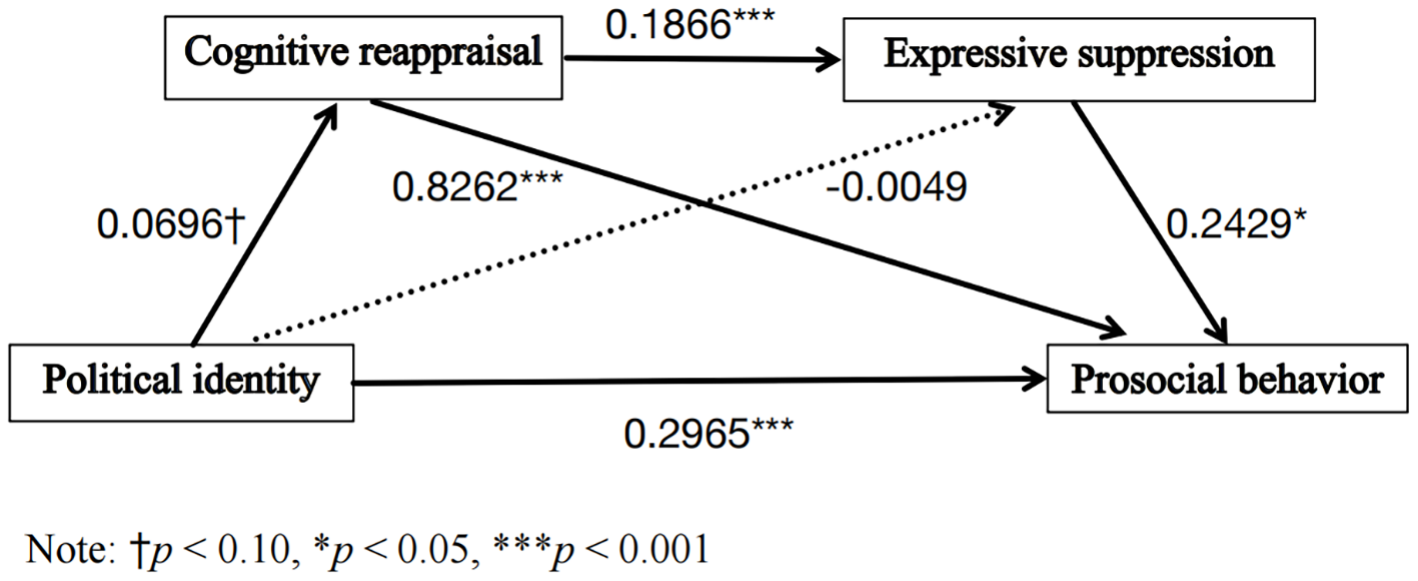
Chain mediation model of political identity on prosocial behavior through cognitive reappraisal and expressive suppression.

Model 6 from the PROCESS macro was used to test the chain mediation effect of political identity on PB via cognitive reappraisal and expressive suppression in the core analysis sample (*N* = 782). The bootstrap method was employed to conduct 5,000 repeated samples, and the 95% confidence intervals for the indirect effects were calculated. The results and path diagram are presented in Table 3 and Figure 1.

**Table 3:** Test of the chain mediation effect of political identity on prosocial behavior (*N* = 782).

Effect type	Unstandardized effect	Boot SE	95%CI
Direct effect	0.2978	0.0739	[0.1527, 0.4428]*
Total indirect effect	0.0596	0.0269	[0.0057, 0.1131]*
Ind1: Political identity → cognitive reappraisal → prosocial behavior	0.0576	0.0249	[0.0089, 0.1092]*
Ind2: Political identity → expressive suppression → prosocial behavior	−0.0011	0.0073	[−0.0158, 0.0143]
Ind3: Political identity → cognitive reappraisal → expressive suppression → prosocial behavior	0.0031	0.0020	[0.0001,0.0079]*

*The 95% Boot CI does not contain 0, indicating a significant effect. The completely standardized effect sizes (indirect effect × SD_X_ / SD_Y_, with SD_X_ = 4.98, SD_Y_ = 11.39) are 0.0252 for Ind1 and 0.0014 for Ind3. The completely standardized direct effect is 0.1306.

Multicollinearity diagnostics indicated that all variance inflation factors (VIFs) ranged from 1.019 to 1.121, which are well below the conventional threshold of 5 and confirm that multicollinearity is not a concern in this model.

The direct effect of political identity on PB was significant [effect = 0.2978, 95% CI (0.1527, 0.4428)]. Regarding indirect effects, the sole mediating effect of cognitive reappraisal was significant [effect = 0.0576, 95% CI (0.0089, 0.1092)], whereas the sole mediating effect of expressive suppression was not significant [effect = −0.0011 95% CI (−0.0158, 0.0143)]. The chained mediation effect of “political identity → cognitive reappraisal → expressive suppression → PB” was significant [effect = 0.0031, 95% CI (0.0001, 0.0079)]. All reported effects are unstandardized; additionally, completely standardized effect sizes for significant indirect effects are provided as a note with Table 3. These results support the chain-mediated role of emotion regulation strategies between political identity and PB. However, does this effect differ among students from different economic backgrounds? To investigate this question, we first conducted a cluster analysis of the sample based on SEA and SES to identify different types of poverty.

### 3.5 Results of cluster analysis of poverty types

Following the clustering procedure described in the Methods Section 2.3, four distinct poverty types were identified based on SEA and SES using the full sample (*N* = 1,070). The resulting cluster centers and sample distributions for the sophomore subsample (*n* = 782) are shown in Table 4. One-way analysis of variance revealed significant differences among the four types in both SEA and SES (*F* = 426.23, 393.65, *ps* < 0.001), indicating the validity of the clustering results.

**Table 4:** Final cluster centers and sample distribution for the four poverty types (*N* = 782).

Poverty types	SES (*M*)	SEA (*M*)	*n* (%)
1. Subjectively Advantaged Type	−0.40	0.71	189 (24.2%)
2. Double-disadvantaged type	−0.81	−0.88	264 (33.8%)
3. Objectively Advantaged Type	0.42	−0.37	209 (26.7%)
4. Dual Affluence Type	1.40	1.22	120 (15.3%)

SES and SEA were both standardized within the full sample (*N* = 1,070). Values are mean *z*-scores for each poverty type.

Based on the objective and subjective economic resource characteristics of each group, the four categories were named as follows:

Subjectively advantaged type: SES is slightly below average and SEA is significantly above average, reflecting positive psychological perception;
Dual-disadvantaged type: Both SEA and SES are significantly below average, indicating a dual scarcity of resources;
Objectively advantaged type: SES is above average but SEA is below average, indicating a discrepancy between subjective perceptions and objective situation;
Dual-affluence type: SES and SEA are both significantly above average, indicating dual resource abundance.

### 3.6 Path analysis and resource effects across different poverty types

Multicollinearity diagnostics for all regression models (based on the variance inflation factor, VIF) indicated that all VIF values ranged from 1.018 to 1.370, well below the conventional threshold of 5. These values confirmed that multicollinearity is not a concern in any of the subgroup analyses.

To further examine differences in the relationships among core variables across the four types, we adopted a stepwise analysis strategy: First, correlations among core variables were tested within each group; second, regression analysis was conducted on variables showing significant correlations to examine their predictive role for PB; finally, based on the regression results, corresponding path models were constructed and tested within each group. The results are presented in Table 5.

**Table 5:** Core pathways and resource effects across four poverty types.

Pathways	Resource effect	Unstandardized effect	Boot SE	95% CI
1. Subjectively advantaged (*n* = 189)				
Subjective affluence → prosocial behavior (direct effect)		0.1253	0.1339	[−0.1388, 0.3894]
Subjective affluence → cognitive reappraisal → prosocial behavior (indirect effect)	Substitution	0.2521	0.0946	[0.0930, 0.4570]*
2. Dual disadvantage (*n* = 264)				
Red music knowledge → prosocial behavior (direct effect)		0.3762	0.2193	[−0.0556, 0.8080]
Red music knowledge → political identity → prosocial behavior (indirect effect)	Compensation	0.0792	0.0508	[0.0050, 0.2001]*
Political identity → prosocial behavior (direct effect)		0.3110	0.1210	[0.0726, 0.5494]*
Cognitive reappraisal → prosocial behavior (direct effect)		1.1185	0.1365	[0.8498, 1.3872]*
3. Objectively advantaged (*n* = 209)				
Political identity → prosocial behavior (direct effect)	Additive (path A)	0.3331	0.1385	[0.0600, 0.6062]*
Cognitive reappraisal → expressive suppression → prosocial behavior (indirect effect)	Additive (path B)	0.2905	0.1003	[0.1134, 0.5036]*
Cognitive reappraisal → prosocial behavior (direct effect)		0.3944	0.1713	[0.0567, 0.7322]*
4. Dual affluence (*n* = 120)				
Cognitive reappraisal → prosocial behavior (direct effect)	Dilution	0.7384	0.1936	[0.3547, 1.1220]*

*The 95% CI does not contain 0, indicating a significant effect.

#### 3.6.1 Subjectively advantaged type

Among subjectively advantaged students (*n* = 189), linear regression was used to test the predictive role of political identity on PB. The effect of political identity was not significant (*p* > 0.05), suggesting that, in this group, political identity is not a direct predictor of PB; its functional role may be substituted by other psychological resources (e.g., cognitive reappraisal). Furthermore, the predictive effects of red music knowledge and expressive suppression on PB were not significant (*p* > 0.05).

Model 4 of the PROCESS macro was used to examine the mediating pathway of subjective affluence–cognitive reappraisal–PB, controlling for gender, student leadership roles, scholarships and grants, household registration, and age. The total effect of subjective affluence on PB was significant (effect = 0.5467, *p* < 0.001). Mediation analysis revealed that the indirect effect of SEA on PB via cognitive reappraisal was significant [effect = 0.2612, 95% CI (0.080, 0.513)], and the direct effect was significant [effect = 0.2855, 95% CI (0.008, 0.563)], indicating that cognitive reappraisal partially mediates the relationship between subjective affluence and PB, accounting for 47.78% of the total effect. Thus, the limited availability of objective resources activates these students’ cognitive reappraisal abilities, thereby promoting PB, demonstrating a resource substitution effect. This finding reveals the mutual substitutability of psychological resources: when one resource (political identity) is insufficient, other psychological resources (subjective affluence and cognitive reappraisal) can perform similar functions.

#### 3.6.2 Dual-disadvantaged type

Among students with dual disadvantages (*n* = 264), linear regression showed that political identity (*β* = 0.143, *p* = 0.011) and cognitive reappraisal (*β* = 0.459, *p* < 0.001) significantly and positively predicted PB, whereas red music knowledge and subjective economic affluence did not significantly predict PB (*ps* > 0.05). Mediation analysis indicated that the mediating effect of cognitive reappraisal between political identity and PB was not significant (*p* > 0.05); however, the indirect effect of red music knowledge on PB via political identity was significant (effect = 0.0792, 95% CI [0.0050, 0.2001]), while the direct effect was not significant (*p* > 0.05), and the total effect was significant (*p* < 0.05), indicating a full mediation model. Thus, for students facing dual resource scarcity, red music knowledge indirectly predicts PB through political identity, reflecting a resource compensatory effect of cultural heritage education. External cultural interventions may thus have an indirect predictive effect on PB by strengthening political identity under conditions of resource scarcity. Within this group, both political identity and cognitive reappraisal directly predicted PB in the regression analyses, and the indirect effect of political identity on PB via cognitive reappraisal was not significant (Bootstrap CI included 0). Therefore, they appear to act as two parallel and independent predictors of PB, rather than being linked in a causal chain.

#### 3.6.3 Objectively advantaged type

Among students with an objectively advantaged type (*n* = 209), two independent pathways were found to additively predict PB. First, political identity had a significant direct effect on PB [effect = 0.3331, 95% CI (0.0600, 0.6062)]. Second, cognitive reappraisal indirectly predicted PB via expressive suppression: the indirect effect was significant [effect = 0.2905, 95% CI (0.1134, 0.5036)], and its direct effect of cognitive reappraisal on PB remained significant [effect = 0.3944, 95% CI (0.0567, 0.7322)]. The chained mediation model (political identity → cognitive reappraisal → expressive suppression → PB) was tested, but its indirect effect was found to be not significant (Bootstrap CI contained 0). The relevant pathways for SEA and red music knowledge were both insignificant (*ps* > 0.05).

These results indicate that political identity and emotion regulation strategies operate independently and additively in the objectively advantaged type, reflecting a resource additive effect. In other words, when objective resources are high but subjective perception is low, multiple psychological resources can act independently to foster PB.

#### 3.6.4 Dual-affluence type

Among students in the dual-affluence group (*n* = 120), only cognitive reappraisal exhibited a direct positive predictive effect on PB [effect = 0.7384, 95% CI (0.3547, 1.1220)]; the pathways associated with political identity, expressive suppression, subjective affluence, and red music knowledge were all insignificant (*ps* > 0.05). This suggests that, for students with dual resource abundance, PB is more directly predicted by their own emotion regulation abilities than by external resources or political identity, reflecting a resource dilution effect. This finding is consistent with the core proposition of COR theory that the influence of a resource is directly proportional to its scarcity.

Synthesizing the above analyses, the four poverty types exhibit qualitative differences in the relationships among political identity, emotion regulation, cultural heritage education, subjective economic affluence, and PB, corresponding, respectively, to four resource effects within conservation of resources theory: resource substitution effect, resource compensation effect, resource additive effect, and resource dilution effect. Together, these four resource effects reveal context-specific variations in psychological functioning across economic resource environments, providing an integrated theoretical framework for understanding the heterogeneous pathways linking political identity to PB.

## 4 Discussion

### 4.1 Political identity and PB: validation of the chain mediation mechanism

We first validated the positive predictive role of political identity on PB across the core sample and found that this relationship is mediated through the chained pathway of “cognitive reappraisal → expressive suppression.” This finding supports an integrative perspective combining social identity theory and emotion regulation theory. As a crucial form of social identity, the sense of belonging and pride inherent in political identity is associated with individuals’ emotion regulation capabilities, which in turn are associated with PB. Specifically, political identity is positively associated with the use of cognitive reappraisal, which may help individuals reinterpret emotional events more flexibly; this greater use of cognitive reappraisal is further associated with reduced reliance on expressive suppression, and this pattern is associated with higher levels of PB. This chained mediation mechanism is consistent with previous research on the mediating role of emotion regulation between social cognition and behavior. Zhang et al. (2023) found that cognitive reappraisal and expressive suppression similarly mediate the relationship between campus living conditions and mental health, further confirming the central role of emotion regulation strategies as psychological transformation mechanisms.

Given the focus on sophomores, the conclusions may apply primarily to this population, and caution is warranted when generalizing the findings to other grade levels. Even after controlling for demographic variables such as gender, student leadership roles, and receipt of scholarships and financial aid, the chained mediation effect remained robust, indicating that political identity has an independent association with PB beyond sociodemographic factors.

### 4.2 Moderating role of poverty types on psychological transformation pathways

A core contribution of this study lies in revealing how poverty types (a combination of SES and SEA) reshape the psychological pathways of political identity. Based on COR theory, we identified four theoretically significant resource effects, each corresponding to a distinct type of impoverished group.

#### 4.2.1 Subjectively advantaged type: resource substitution effect

Among subjectively advantaged students, the political identity pathway did not hold; instead, SEA positively predicted PB through cognitive reappraisal, demonstrating a resource substitution effect. These students have limited objective resources, but through positive subjective perceptions, SEA itself becomes a psychological resource. Through its positive association with cognitive reappraisal, it may serve a function similar to political identity. This finding suggests the substitutability of psychological resources; in other words, when one resource is insufficient, other psychological resources can perform similar functions. This embodies the principle of functional equivalence of resources, whereby different resources can serve similar psychological functions (Diener and Fujita, 1995). As pointed out by resilience and broaden-and-build theories, positive psychological perception can play a protective role in adversity, compensate for missing external resources, and further explain how positive emotions and positive perception construct lasting personal resources, thereby generating substitutive benefits (Masten, 2001).

#### 4.2.2 Dual-disadvantaged type: resource compensation effect

Among students with dual disadvantage, the chain mediation pathway of political identity did not hold; however, red music knowledge indirectly predicted PB through political identity, demonstrating a resource compensation effect, while emotion regulation ability was found to be an independent facilitating factor. Thus, for students facing a dual shortage of objective and subjective resources, external cultural resources may play a compensatory role through association with internal psychological resources. When individuals lack direct economic and psychological resources, cultural symbolic resources can act as alternative resources that are associated with filling the resource gap. This result aligns with the core proposition of COR theory that “resource loss drives resource acquisition”, while simultaneously extending its scope from individual to cultural symbolic resources, providing a theoretical basis for cultural interventions targeting vulnerable groups. This conclusion also resonates with the stress-buffering model of social support: external resources such as social support do not always yield universal benefits, but rather exert compensatory and protective effects by meeting specific needs when individuals face stress (Cohen and Wills, 1985). Consequently, for resource-poor and disadvantaged groups, the compensatory function of external cultural resources may take precedence over the conventional integration pathways of internal psychological resources. This further corroborates the core tenet of COR theory: the potency of a resource is directly proportional to its scarcity, and the impact of external resources is most pronounced when the recipient’s own resources are most depleted.

#### 4.2.3 Objectively advantaged type: resource additive effect

Among objectively advantaged students, two independent pathways—political identity and emotion regulation—additively predicted PB. This pattern reflects a resource additive effect rather than a chained integration. Although these students possess adequate objective resources, their subjective sense of abundance is low, resulting in a state of dissonance between subjective and objective perceptions. In such a state, multiple psychological resources (political identity and emotion regulation strategies) appear to operate independently and additively to foster PB. This finding suggests that when objective resources do not align with subjective perceptions, individuals may rely on several independent pathways rather than a single integrated chain. This result is consistent with relative deprivation theory, which posits that the gap between subjective perception and objective reality may trigger multiple psychological adjustments. Furthermore, cognitive dissonance theory (Festinger, 1957) also suggests that incongruence can motivate psychological efforts, which in this case may manifest as additive rather than sequential pathways.

#### 4.2.4 Dual-affluence type: resource dilution effect

Among students with dual affluence, only cognitive reappraisal directly predicted PB, while political identity did not show a significant association, reflecting the dilution effect of resources. The resource dilution hypothesis suggests that when both objective and subjective resources are abundant, political identity, as one of many resources, is diluted by the abundant resources, and PB was directly associated with an individual’s emotion regulation ability. This hypothesis was initially used to explain the distribution of family resources among children (Blake, 1981), and it has since been extended to a broader social context (Downey, 1995). When individuals have abundant resource reserves, the marginal contribution of a single resource (e.g., political identity) to behavior decreases, and behavior is more likely to stem from stable personal traits (e.g., emotion regulation ability) (Lazarus and Folkman, 1984). This is consistent with the core proposition of COR theory: the intensity of resource utilization is proportional to the scarcity of resources. When resources are abundant, the importance of a single resource decreases; when resources are scarce, their value is amplified. This discovery also echoes the basic viewpoint of Maslow’s hierarchy of needs theory: when basic needs are met, higher-level psychological needs dominate behavior.

### 4.3 Group-specific effects of cultural heritage education

We found that knowledge of red music—a key form of cultural heritage education in China—indirectly predicted PB through political identity only among the “dual-disadvantaged” type, while it was not significant in other types. This group-specific finding has significant theoretical implications: cultural heritage education may not be a universally effective intervention; its association with PB was primarily observed among groups most in need of resource compensation. For students facing dual resource deprivation, cultural heritage education may provide an alternative psychological resource by strengthening political identity, potentially acting as a “lifeline in times of need” rather than merely “adding icing on the cake.” This result is highly consistent with the predictions of COR theory—the effects of external resource interventions are most pronounced when recipients are most resource-deprived. It further suggests that future research must consider group heterogeneity when evaluating the effectiveness of educational interventions to avoid letting “average effects” obscure important group differences.

### 4.4 Theoretical contributions

First, the study reveals the chained mediation mechanism through which political identity is associated with PB. By incorporating emotion regulation into the analytical framework, this study opens the “black box” between political identity and PB, providing a process-oriented explanation for how identity relates to action. This finding bridges social identity theory, emotion regulation theory, and PB research, thus promoting integration across theoretical domains.

Second, the study introduced the concept of “poverty type” and validated its moderating role. By combining SES with subjective feelings of affluence, we identified four types with distinct resource characteristics and revealed qualitative differences in the psychological pathways of political identity’s association across these types. This classification transcends the linear “high/low” thinking found in traditional research, providing a new analytical unit for understanding the heterogeneity of the relationship between social class and PB, and responding to the theoretical calls for effect heterogeneity in recent meta-analyses.

Finally, the study expands the application boundaries of COR theory. By identifying four resource effects—substitution, compensation, additive, and dilution—this research extends the scope of COR theory from individual resources to collective symbolic resources and subjectively perceived resources, thereby enriching its conceptual framework. Additionally, it provides a new conceptual tool for understanding the dynamic operation mechanisms of resources.

Crucially, the study clarifies that poverty moderates the political identity–PB link through two distinct pathways: structural (material constraints captured by SES) and psychological (scarcity-induced cognitive shifts captured by SEA). The four poverty types identified in this research represent different combinations of these two pathways, which may help to reconcile contradictory findings in the literature on social class and PB and offer a more coherent perspective.

In summary, all proposed hypotheses received support. Hypothesis 1 was confirmed: political identity was found to be associated with PB through the chain mediation of cognitive reappraisal and expressive suppression. Hypotheses 2a–2d were also supported, showing that the four poverty types exhibit distinct pathways corresponding to substitution, compensation, additive, and dilution effects, respectively. Hypothesis 3 received exploratory support, as red music knowledge indirectly predicted PB only in the dual-disadvantaged type.

### 4.5 Implications for practice

First, fostering political identity should prioritize the concurrent development of emotion regulation skills. We found that political identity is associated with PB through the chain of mediating effects involving cognitive reappraisal and expressive suppression, suggesting that emotion regulation is a key psychological pathway for political identity to fulfill its positive social functions. Therefore, while fostering students’ political identity, emotion regulation training should be conducted concurrently to help students master adaptive strategies such as cognitive reappraisal and reduce their reliance on expressive suppression, thereby more effectively relating internal identity to external PB. This “identity−emotion” synergistic cultivation model offers new insights for the deep integration of ideological and political education with mental health education in higher education institutions.

Second, accurately identifying students’ resource types is a prerequisite for effective intervention. Research reveals qualitative differences in the psychological transformation pathways of students with different types of poverty. This requires educators to move beyond “one-size-fits-all” intervention models and, based on students’ objective economic circumstances and subjective psychological perceptions, accurately identify their resource types to implement differentiated interventions. Specifically, for students with dual disadvantages, the focus should be on strengthening the compensatory role of cultural education (e.g., red music knowledge) to indirectly encourage PB by enhancing political identity; for objectively advantaged students, emphasis should be placed on both directly fostering political identity and cultivating emotion regulation skills (cognitive reappraisal and expressive suppression) to help them bridge the gap between objective and subjective perceptions, with these two pathways working in parallel without conflict; for subjectively advantaged students, their existing positive perceptions should be reinforced by guiding them to channel their subjective sense of abundance into prosocial motivation; for students with dual affluence, the cultivation of emotion regulation skills in itself can encourage PB.

Third, cultural heritage education should focus on the groups most in need of resource compensation. The current findings indicate that red music knowledge is significantly associated with PB only among students with dual disadvantages, suggesting that, given limited resources, cultural education resources should be precisely targeted at students experiencing a dual shortage of both objective and subjective resources to leverage their resource-compensating function and maximize educational benefits. This provides empirical evidence for the optimal allocation of educational resources.

Fourth, the intervention value of subjective psychological perceptions should be emphasized. We found that a sense of subjective affluence is positively associated with PB through cognitive reappraisal in subjectively advantaged students, suggesting that enhancing students’ positive economic perceptions through psychological counseling and cognitive restructuring may generate positive psychological effects even when objective resources remain unchanged. This finding offers new insights for the synergistic development of mental health and values education.

## 5 Limitations and future directions

First, regarding the cross-sectional design and sample, although the chained mediation analysis is based on theoretical derivation, cross-sectional data cannot confirm causal relationships; therefore, the mediation effects reported in this study should be interpreted as correlational patterns consistent with the theoretical model and not as causal evidence. Simultaneously, to control for grade-level effects, we included only sophomore medical students; therefore, conclusions should be generalized to other grade levels and majors with caution. Future research should adopt longitudinal tracking or experimental designs to test causal relationships and include multi-grade, multi-major samples to examine the generalizability of the findings.

Second, regarding measurement dimensions and exploratory findings, we focused solely on knowledge of red music and did not comprehensively cover the rich connotations of cultural heritage education; furthermore, the “resource substitution effect” identified in the subjectively advantaged subtype stems from exploratory analysis, and its robustness remains to be verified. Future research could incorporate additional dimensions, such as institutional history and cultural role models, and employ larger samples or experimental designs to specifically test the compensatory protective role of subjective perceptions in resource-scarce contexts.

Third, regarding the construction and stability of poverty types, it is worth noting that cluster solutions are inherently sample-dependent, and their stability and generalizability require further validation. Although we obtained the poverty typology from the full sample (*N* = 1,070), the specific cluster structure may still vary according to sample composition. Future research should employ larger and more diverse samples to further test the robustness of the poverty-type structure and explore its dynamic changes over time.

Fourth, regarding macro-level sociocultural factors, we did not include macro-level factors such as school atmosphere, social support, and regional culture. Future research could employ multilevel linear models to examine the moderating effects of school-level factors on individual psychological transformation pathways.

## Data Availability

The raw data supporting the conclusions of this article will be made available by the authors, without undue reservation.
