# Supplementary material for: How do poverty types reshape the effect of political identity on prosocial behavior? The chain mediation role of emotion regulation and compensatory effect of cultural heritage education
Source: Front Psychol. 2026 May 15;17:1849695. doi: 10.3389/fpsyg.2026.1849695 (PMC13219352; doi:10.3389/fpsyg.2026.1849695)
Supplement: Supplementary file 1 [file Supplementary_file_1.DOCX]

In the Methods (Section 2.2), all scales/dimensions show acceptable to excellent reliability and convergent validity (*α* ≥ 0.77, *ω* ≥ 0.77, AVE ≥ 0.50 except SEA = 0.499, CR ≥ 0.83). For SEA, the slightly lower AVE is explained by the presence of six reverse‑coded items and the high CR (0.908), and convergent validity is still considered acceptable.

Table S1. Reliability and validity of scales and dimensions

| Scale / Dimension | Items | Cronbach’s *α* | McDonald’s *ω* | AVE | CR |
| --- | --- | --- | --- | --- | --- |
| Political Identity (total) | 17 | 0.943 | 0.942 | – | – |
| - Nation & ethnicity | 8 | 0.846 | 0.856 | 0.794 | 0.969 |
| - Political system & values | 9 | 0.949 | 0.950 | 0.900 | 0.988 |
| Emotion Regulation |  |  |  |  |  |
| - Cognitive reappraisal | 6 | 0.893 | 0.893 | 0.658 | 0.919 |
| - Expressive suppression | 4 | 0.813 | 0.815 | 0.553 | 0.831 |
| Prosocial Behavior (total) | 26 | 0.946 | 0.946 | – | – |
| - Openness | 4 | 0.790 | 0.793 | 0.587 | 0.849 |
| - Anonymity | 5 | 0.840 | 0.839 | 0.645 | 0.900 |
| - Altruism | 4 | 0.823 | 0.825 | 0.701 | 0.903 |
| - Compliance | 5 | 0.811 | 0.816 | 0.598 | 0.881 |
| - Emotionality | 5 | 0.851 | 0.855 | 0.664 | 0.907 |
| - Urgency | 3 | 0.766 | 0.767 | 0.676 | 0.862 |
| SEA | 10 | 0.888 | 0.889 | 0.499 | 0.908 |
| Red Music Knowledge | 12 | 0.818 | – | – | – |

Note: Red music knowledge items are binary; AVE and CR are not applicable. SES is a composite score and is not included in the table.

In the Statistical Analysis Strategy (Section 2.3), as a robustness check, we performed LPA on the same standardized SES and SEA variables in the core sample (*n* = 782). The LPA results (Tables S2 and S3 below) closely matched the K‑means typology (three of four classes matched).

Table S2. Fit indices for LPA models (2–4 classes, *n* = 782)

| Classes | AIC | BIC | Entropy | LMR-LRT (*p*) |
| --- | --- | --- | --- | --- |
| 2 | 4271.70 | 4304.33 | 0.869 | <0.001 |
| 3 | 4254.19 | 4300.81 | 0.607 | 0.276 |
| 4 | 4236.39 | 4296.99 | 0.798 | 0.033 |

Table S3. Mean z‑scores of the 4‑class LPA solution (*n* = 782)

| Class | SES (mean) | SEA (mean) | Corresponding K‑means type |
| --- | --- | --- | --- |
| 1 | -0.61 | -2.05 | Dual‑disadvantaged |
| 2 | 1.87 | 0.61 | Dual‑affluent |
| 3 | -0.39 | -0.24 | (no direct match) |
| 4 | 0.15 | 1.41 | Subjectively advantaged |
